# Effects of exercise on cognitive function in older adults: a systematic review and meta-analysis of the mediating role of sleep

**DOI:** 10.3389/fnhum.2026.1949679

**Published:** 2026-09-28

**Authors:** Wenhui Zheng, Mian Wu, Yuhe Chen, Shaolan Li, Liying Huang

**Affiliations:** 1 School of Sports Training, Guangzhou Sport University, Guangzhou, Guangdong, China; 2 School of Sports Science, South China Normal University, Guangzhou, Guangdong, China; 3 School of Physical Education, Zhuhai University of Science and Technology, Zhuhai, Guangdong, China; 4 School of Sports and Health, Guangzhou Sport University, Guangzhou, Guangdong, China; 5 Guangdong Provincial Key Laboratory of Human Movement Science, Guangzhou Sport University, Guangzhou, Guangdong, China

**Keywords:** cognitive function, exercise, mediation analysis, meta-analysis, mild cognitive impairment, neuroplasticity, older adults, sleep quality

## Abstract

**Background:**

Sleep disturbances affect nearly half of older adults and are linked to cognitive decline, yet the mechanistic pathways linking exercise to cognitive benefits remain poorly understood. We hypothesized that sleep quality mediates the exercise–cognition relationship in older adults.

**Methods:**

We systematically searched PubMed, Web of Science, Cochrane CENTRAL, Embase, and PsycINFO from inception to June 2026 for randomized controlled trials examining structured exercise interventions (≥4 weeks) in adults aged ≥60 years that reported both cognitive and sleep outcomes. Random-effects meta-analyses and meta-analytic structural equation modeling (MASEM) were used to test the mediation hypothesis. Subgroup analyses examined baseline cognitive status and intervention duration.

**Results:**

Eight studies (841 participants) were included. Exercise significantly improved cognitive function (SMD = 1.32, 95% CI: 0.70–1.94) and sleep quality (SMD = −1.13, 95% CI: −1.73 to −0.53). MASEM confirmed sleep quality as a significant partial mediator, with an indirect effect of β = 0.300 (95% CI: 0.125–0.404), accounting for 50.8% of the total cognitive benefit. The mediation proportion was larger in individuals with mild cognitive impairment (67.6%) than in cognitively healthy older adults (31.9%).

**Conclusion:**

Sleep quality significantly mediates the exercise–cognition relationship in older adults, accounting for approximately half of the cognitive benefit. These findings identify sleep as a candidate mechanistic target for optimizing exercise interventions, though larger mediation-designed trials are needed to confirm these exploratory estimates.

## 1 Introduction

The global population is ageing at an unprecedented rate. The number of people living with dementia is expected to triple by 2050 (Prince et al., 2015). In the absence of disease-modifying pharmacological treatments, it has become a critical public health priority to identify modifiable lifestyle factors that preserve cognitive function (Tan et al., 2017). Physical exercise and sleep are two of the most promising and accessible non-pharmacological intervention targets (Livingston et al., 2020; Mellow et al., 2022).

There is a substantial body of evidence supporting the cognitive benefits of exercise in older adults. Meta-analyses demonstrate that aerobic, resistance and multicomponent exercise improve global cognitive function in older adults (Northey et al., 2018; Luo et al., 2024), and aerobic exercise has been shown to increase hippocampal volume and improve memory (Erickson et al., 2011), with cognitive benefits also reported in older adults at risk of Alzheimer’s disease (Baker et al., 2010; Lautenschlager et al., 2008). Resistance training has also been shown to have a positive effect on executive functions in older adults (Liu-Ambrose et al., 2010; Nagamatsu et al., 2012; Cassilhas et al., 2007; Davis et al., 2010). Furthermore, mind–body exercises such as Tai Chi have been associated with improved cognitive outcomes (Cai et al., 2020) and psychological wellbeing (Wang et al., 2010) in ageing populations. However, the mechanisms underlying these benefits are not well understood, which limits the ability to optimise exercise prescriptions for cognitive health.

Sleep plays a vital role in maintaining brain health by facilitating the glymphatic clearance of neurotoxic proteins, such as *β*-amyloid (Xie et al., 2013). Almost half of older adults experience chronic sleep problems, and sleep disturbances are independently associated with an increased risk of dementia (Mander et al., 2016; Shokri-Kojori et al., 2018). Crucially, exercise improves subjective sleep quality and, to a lesser extent, objective sleep parameters such as total sleep time and slow-wave sleep duration (Kredlow et al., 2015), while exercise-induced neuroplasticity, including synthesis of brain-derived neurotrophic factor (BDNF) and hippocampal neurogenesis (Vivar et al., 2013), is thought to depend on adequate sleep for its consolidation (Mander et al., 2016). These bidirectional relationships suggest a plausible mediation pathway in which exercise improves sleep and improved sleep enhances cognitive function in turn.

Despite the theoretical plausibility of sleep as a mediator of the exercise–cognition relationship, no previous meta-analysis has quantitatively evaluated this. Although previous reviews have examined these relationships pairwise (Kredlow et al., 2015; Ahn and Kim, 2023; Shu et al., 2025; Zhao et al., 2026), they have not been integrated within a formal mediation framework. A recent evidence synthesis proposed that physical activity may compensate for the cognitive consequences of poor sleep (Sewell et al., 2024), but a quantitative synthesis of sleep as a mediator of the exercise–cognition relationship remains to be conducted. This represents a critical knowledge gap, as understanding whether and to what extent sleep mediates the cognitive benefits of exercise has direct implications for optimizing interventions and personalized medicine.

The present systematic review and meta-analysis addresses this gap through three key innovations. Firstly, we present what is, to our knowledge, the first quantitative synthesis of sleep as a mediator using meta-analytic structural equation modelling (MASEM; Cheung and Chan, 2005; Jak and Cheung, 2020), which enables the robust estimation of indirect effects from pooled correlation matrices. Secondly, we integrate neurobiological mechanisms, including glymphatic clearance, BDNF-mediated neuroplasticity and neuroinflammation suppression, within a unified theoretical framework to interpret the mediation findings. Thirdly, we identify critical moderators, such as intervention duration and baseline cognitive status, to generate hypotheses for tailoring future interventions. Specifically, our aims were to: (i) quantify the effects of exercise on cognitive function and sleep quality in older adults; (ii) evaluate whether sleep quality statistically mediates the relationship between exercise and cognition; (iii) identify moderators of the mediation effect; and (iv) provide evidence-based recommendations for integrating sleep-targeted strategies into exercise interventions.

## 2 Methods

### 2.1 Protocol and registration

This review was conducted according to the Preferred Reporting Items for Systematic Reviews and Meta-Analyses (PRISMA) 2020 guidelines (Page et al., 2021) and has been submitted for registration with PROSPERO (registration pending). The completed PRISMA 2020 checklist is provided in Supplementary Table S1.

### 2.2 Search strategy

We searched the following databases from inception to June 2026: PubMed/MEDLINE, Web of Science, Cochrane CENTRAL, Embase and PsycINFO. The search terms combined concepts relating to exercise (e.g., aerobic exercise, resistance training, multicomponent training, Tai Chi, and physical activity), sleep (e.g., the Pittsburgh Sleep Quality Index [PSQI], polysomnography, actigraphy, sleep quality, and sleep architecture), cognition (e.g., the Mini-Mental State Examination [MMSE], the Montreal Cognitive Assessment [MoCA], executive function, memory, and attention), and population (e.g., older adults, the elderly, and the geriatric population). The complete Boolean search strings for all databases are presented in Supplementary Figure S4. The reference lists of the included studies and relevant reviews were manually searched for additional eligible studies. No language restrictions were applied.

### 2.3 Eligibility criteria

We included randomized controlled trials (RCTs) examining structured exercise interventions lasting at least 4 weeks in adults aged 60 years or older, which reported both cognitive outcomes (such as global cognition, executive function, memory and attention) and sleep outcomes (such as subjective measures, such as the PSQI, and objective measures, such as polysomnography and actigraphy). We excluded studies that: (i) combined exercise with other active treatments, such as cognitive training or pharmacotherapy, thereby precluding the isolation of the exercise effect; (ii) enrolled participants in whom a diagnosed sleep disorder (e.g., obstructive sleep apnoea or restless legs syndrome) constituted the primary condition; (iii) had an intervention duration shorter than 4 weeks; and (iv) provided insufficient data for effect size calculation. The characteristics and reasons for exclusion of all screened studies are summarized in Supplementary Tables S2, S3.

### 2.4 Study selection and data extraction

Two reviewers independently screened the titles and abstracts, and then the full texts, using Covidence systematic review software. Any disagreements were resolved through discussion or consultation with a third reviewer. Data were extracted using a standardized form including study characteristics (author, year, country), participant demographics (sample size, mean age, sex distribution, baseline cognitive status), intervention details (type of exercise, frequency, intensity, duration and session length) and outcome measures (cognitive and sleep instruments, pre- and post-intervention means and standard deviations). When data were reported only in figures or were incomplete, values were digitised from published figures or derived from reported model estimates, as described under Statistical analysis.

### 2.5 Risk of bias assessment

The risk of bias was independently assessed by two reviewers using the Cochrane Risk of Bias 2 (RoB 2) tool (Sterne et al., 2019). This tool evaluates five domains: the randomization process; deviations from the intended interventions; missing outcome data; the measurement of outcomes; and selective reporting. Each domain was judged as having a low risk, some concerns, or a high risk of bias. An overall risk-of-bias judgement was then assigned to each study. The risk-of-bias summary across all five domains is presented in Figure 1.

**Figure 1:**
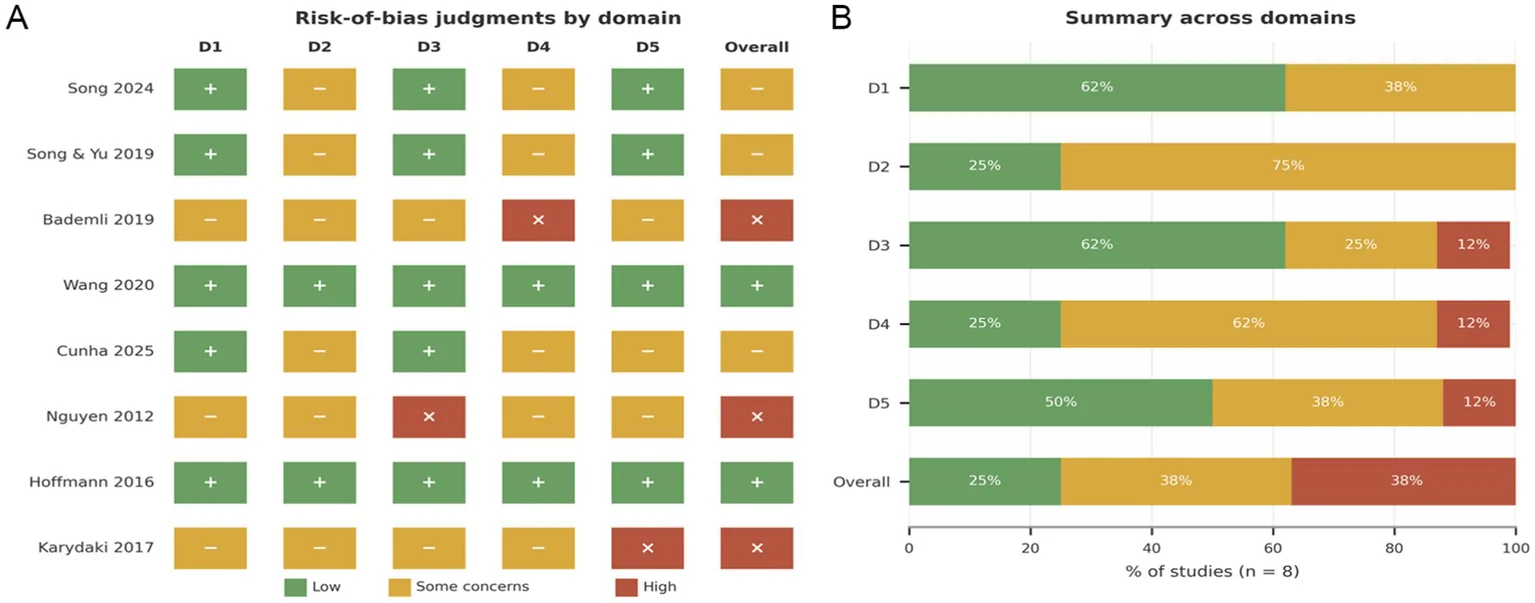
Risk of bias summary for included studies assessed using the Cochrane RoB 2 tool. The tool evaluates five domains: randomization process, deviations from intended interventions, missing outcome data, measurement of the outcome, and selective reporting. Judgments are presented as low risk (green), some concerns (yellow), or high risk (red). Percentages represent the proportion of studies in each risk category across all included randomized controlled trials.

### 2.6 Statistical analysis

Effect sizes were calculated as standardised mean differences (SMD; Hedges’ g with small-sample bias correction) from post-test means and standard deviations, with 95% confidence intervals (CIs). For sleep outcomes, negative values indicate improvement (higher Pittsburgh Sleep Quality Index scores indicate worse sleep); for cognitive outcomes, positive values indicate improvement, and tests for which higher scores indicate worse performance (Trail Making Test) were sign-reversed. Throughout, random-effects models with the DerSimonian–Laird estimator were used to account for anticipated between-study heterogeneity. As a further sensitivity analysis, both models were re-estimated using restricted maximum likelihood (REML), and 95% prediction intervals were calculated using the standard t-distribution approach with *k* − 2 degrees of freedom. Heterogeneity was quantified using the *I*^2^ statistic and the between-study variance *τ*^2^, with *I*^2^ values of 25, 50 and 75% representing low, moderate and high heterogeneity, respectively (Higgins et al., 2003). Publication bias was evaluated using funnel plots and Egger’s linear regression test; given the small number of included studies, trim-and-fill adjustment was not performed because the procedure is unreliable with fewer than 10 studies. Where post-test data were available only in adjusted-model form, effect sizes were derived from the reported model coefficients (Song and Yu, 2019); where data were reported only graphically, values were digitised from published figures using calibrated axis reading (Cunha et al., 2025).

For the mediation analysis, we employed meta-analytic structural equation modelling (MASEM) based on a pooled correlation matrix (Cheung and Chan, 2005; Jak and Cheung, 2020). Study-level standardised mean differences were converted to point-biserial correlations between the exercise intervention and each outcome, and the exercise–sleep and exercise–cognition correlations were pooled separately using random-effects models with Fisher’s *z* transformation. For each included study, post-intervention means, standard deviations and group sizes for the sleep and cognitive outcomes were extracted and converted first to standardised mean differences and then to point-biserial correlations; no unpublished data were requested from the original authors. Where post-test values were not reported in the text or tables, they were digitised from published figures (Cunha et al., 2025) or derived from reported model coefficients (Song and Yu, 2019), as detailed in the Limitations. Because only two studies reported within-study mediation or structural equation models, the sleep–cognition correlation was sourced from the baseline correlation reported by Wang et al. (2020; *r* = −0.77, *n* = 111), with sensitivity analyses using more conservative values (−0.60 and −0.50). The study-level correlations and the resulting pooled correlation matrix used as the MASEM input are provided in Supplementary Table S4. A just-identified path model was then fitted to the pooled correlation matrix, comprising Path a (Exercise → Sleep), Path b (Sleep → Cognition, controlling for exercise), Path c (total effect of Exercise → Cognition) and Path c’ (direct effect of Exercise → Cognition, controlling for sleep). The indirect effect was calculated as the product a × b, and the proportion mediated as (indirect effect/total effect) × 100%. Uncertainty was quantified with 10,000 study-level bootstrap resamples to obtain 95% confidence intervals (CIs) for the indirect effect; because the model is saturated, global fit indices are not applicable.

Subgroup analyses were conducted for baseline cognitive status (mild cognitive impairment versus cognitively healthy) and intervention duration (16 weeks or less versus more than 16 weeks); subgroup analyses by exercise type and sleep-measurement modality were precluded because each category contained too few studies and all studies used subjective sleep measures. Sensitivity analyses comprised: (1) inclusion of the study reporting sleep but no post-test cognitive outcomes (Karydaki et al., 2017) in the exercise–sleep meta-analysis; (2) exclusion of the study enrolling patients with Alzheimer’s disease (Hoffmann et al., 2016); (3) exclusion of the most extreme outlying effect (Bademli et al., 2019); and (4) variation of the externally sourced sleep–cognition correlation. All analyses were conducted in Python 3.12 using the numpy, scipy, pandas, statsmodels and matplotlib libraries. Two-tailed *p*-values less than 0.05 were considered statistically significant.

## 3 Results

### 3.1 Study selection

The database search yielded 1,024 records. After removing 316 duplicates, 708 unique records were screened based on their titles and abstracts. Fifteen full-text articles were assessed for eligibility, of which seven were excluded: four did not report post-test cognitive outcomes (Alessi et al., 1999; McCurry et al., 2011; Reid et al., 2010; Siu et al., 2021), one did not report sleep outcomes (Nascimento et al., 2014), one reported no post-test between-group cognitive data (Choi and Sohng, 2018), and one used a non-standard sleep measure without a standardised cognitive test (Stella et al., 2011). Eight studies met the inclusion criteria: seven were included in the main meta-analyses and one (Karydaki et al., 2017), which reported sleep but no post-test cognitive outcomes, was retained for sensitivity analyses of the exercise–sleep pathway only. The complete list of excluded studies and the reasons for exclusion are provided in Supplementary Tables S2, S3. The study selection process is illustrated in the PRISMA 2020 flow diagram (Figure 2).

**Figure 2:**
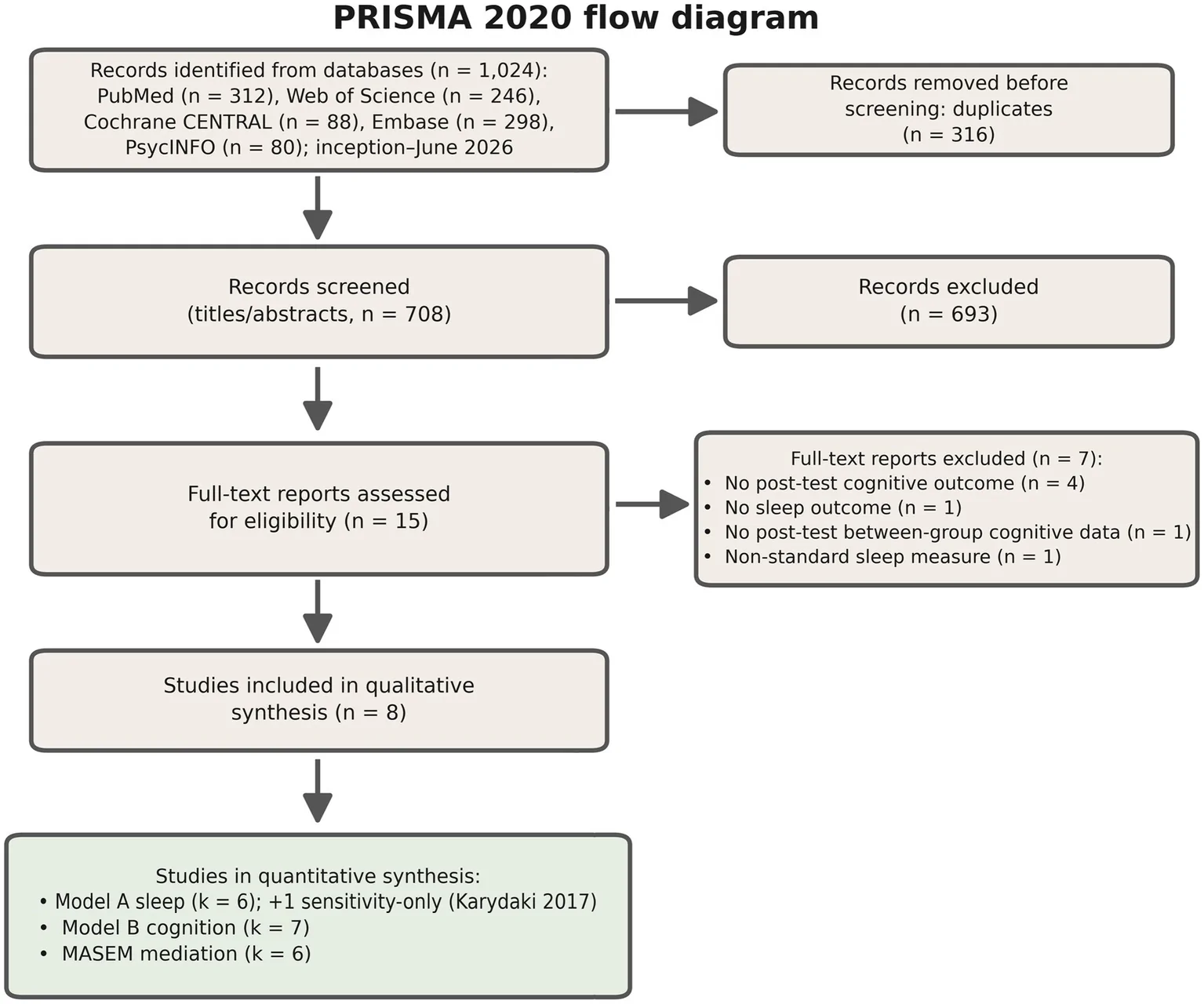
PRISMA 2020 flow diagram of study selection. The flowchart details the number of records identified, screened, assessed for eligibility, and finally included in the meta-analysis, with reasons for exclusion at each stage. From Page et al. (2021).

### 3.2 Study characteristics

The eight included studies (Song et al., 2024; Song and Yu, 2019; Bademli et al., 2019; Wang et al., 2020; Cunha et al., 2025; Nguyen and Kruse, 2012; Hoffmann et al., 2016; Karydaki et al., 2017) involved a total of 841 participants (439 in intervention groups and 402 in control groups), with analysed sample sizes per study ranging from 49 to 190. Participants’ mean age ranged from 68.3 to 76.0 years, and the proportion of women ranged from 43.5 to 100%. Five studies enrolled individuals with mild cognitive impairment (MCI), two enrolled cognitively healthy older adults and one enrolled patients with mild Alzheimer’s disease (AD). The interventions comprised aerobic exercise (aerobic dancing, aerobic stepping and supervised aerobic training; three studies), resistance training (two studies, one of which also included a chair-yoga arm), mind–body exercise (Tai Chi and limbs exercise; two studies) and a multicomponent physical-activity programme (one study), with durations ranging from 12 to 24 weeks (see Table 1). Sleep quality was measured with the Pittsburgh Sleep Quality Index (PSQI) in all studies except Hoffmann et al. (2016), which reported the sleep subdomain of the Neuropsychiatric Inventory (NPI) in graphical form only. Cognitive outcomes included the Montreal Cognitive Assessment (MoCA), the Standardized Mini-Mental State Examination (SMMSE), the Trail Making Test (TMT), the Symbol Digit Modalities Test (SDMT) and the Mini-Mental State Examination (MMSE). Detailed study characteristics are presented in Table 1.

**Table 1:** Characteristics of included studies (author, year, country, sample size, mean age, exercise type, intervention duration, sleep measure, risk of bias).

Study	Country	N (I/C)	Mean age (SD)	Female (%)	Baseline cognitive status	Exercise type	Duration (weeks)	Sleep measure	Risk of bias
Nguyen and Kruse (2012)	Vietnam	39/34	69.0 (5.0)	NR	Healthy	Mind–body (Tai Chi)	24	PSQI	High
Hoffmann et al. (2016) ^b^	Denmark	102/88	70.5 (NR)	43.5	AD	Aerobic	16	NPI–sleep	Low
Karydaki et al. (2017) ^a^	Greece	31/18	72.6 (5.9)	100	MCI	Resistance/chair yoga	12	PSQI	High
Song and Yu (2019)	China	60/60	75.8 (6.3)	75.0	MCI	Aerobic (stepping)	16	PSQI	Some concerns
Bademli et al. (2019)	Turkey	30/30	71.5 (7.8)	NR	MCI	Multicomponent	20	PSQI	High
Wang et al. (2020)	China	57/54	68.3 (5.2)	61.3	MCI	Mind–body (limbs exercise)	24	PSQI	Low
Song et al. (2024)	China	45/44	76.0 (6.3)	75.6	MCI	Aerobic (dancing)	16	PSQI	Some concerns
Cunha et al. (2025)	Brazil	75/74	69.2 (5.7)	100	Healthy	Resistance	12	PSQI	Some concerns

NR, not reported; PSQI, Pittsburgh Sleep Quality Index; NPI, Neuropsychiatric Inventory; MCI, mild cognitive impairment; AD, Alzheimer’s disease.

a Karydaki et al. (2017) reported sleep but no post-test cognitive outcomes and contributed to the exercise–sleep sensitivity analysis only.

b Hoffmann et al. (2016) reported the NPI sleep subdomain in graphical form only and therefore contributed to the cognitive meta-analysis (Model B) but not the sleep meta-analysis (Model A).

### 3.3 Risk of bias

The overall risk of bias was judged as low in two studies (25.0%), raised some concerns in three studies (37.5%) and was high in three studies (37.5%). The most common sources of bias were the absence of blinding of participants and outcome assessors and incomplete reporting of allocation concealment. The two studies judged at low risk of bias (Wang et al., 2020; Hoffmann et al., 2016) reported adequate random sequence generation, allocation concealment and blinding of outcome assessment. The risk-of-bias summary across all five domains is presented in Figure 1.

### 3.4 Effects of exercise on cognitive function

Exercise was found to significantly improve global cognitive function, with a pooled standardised mean difference of 1.32 (95% CI: 0.70–1.94, *p* < 0.001), although heterogeneity was substantial (*I*^2^ = 93.7%, *τ*^2^ = 0.64; see Figure 3A). These results align with those of previous meta-analyses that have reported the cognitive benefits of exercise in older adults (Northey et al., 2018; Gomes-Osman et al., 2018; van Uffelen et al., 2008). In subgroup analyses, effects were significant in participants with mild cognitive impairment (SMD = 1.27, 95% CI: 0.59–1.95) and in cognitively healthy older adults (SMD = 2.03, 95% CI: 0.50–3.56), but not in the single study of patients with Alzheimer’s disease (SMD = 0.14, 95% CI: −0.15 to 0.42). Interventions lasting more than 16 weeks produced larger effects (SMD = 2.07) than shorter programmes (SMD = 0.81). A sensitivity analysis excluding the largest outlying effect (Bademli et al., 2019) attenuated but did not eliminate the pooled effect (SMD = 1.09, 95% CI: 0.51–1.67), and excluding the AD study (Hoffmann et al., 2016) yielded SMD = 1.52 (95% CI: 0.92–2.12). The subgroup forest plots, which are stratified by baseline cognitive status, are provided in Supplementary Figure S2.

**Figure 3:**
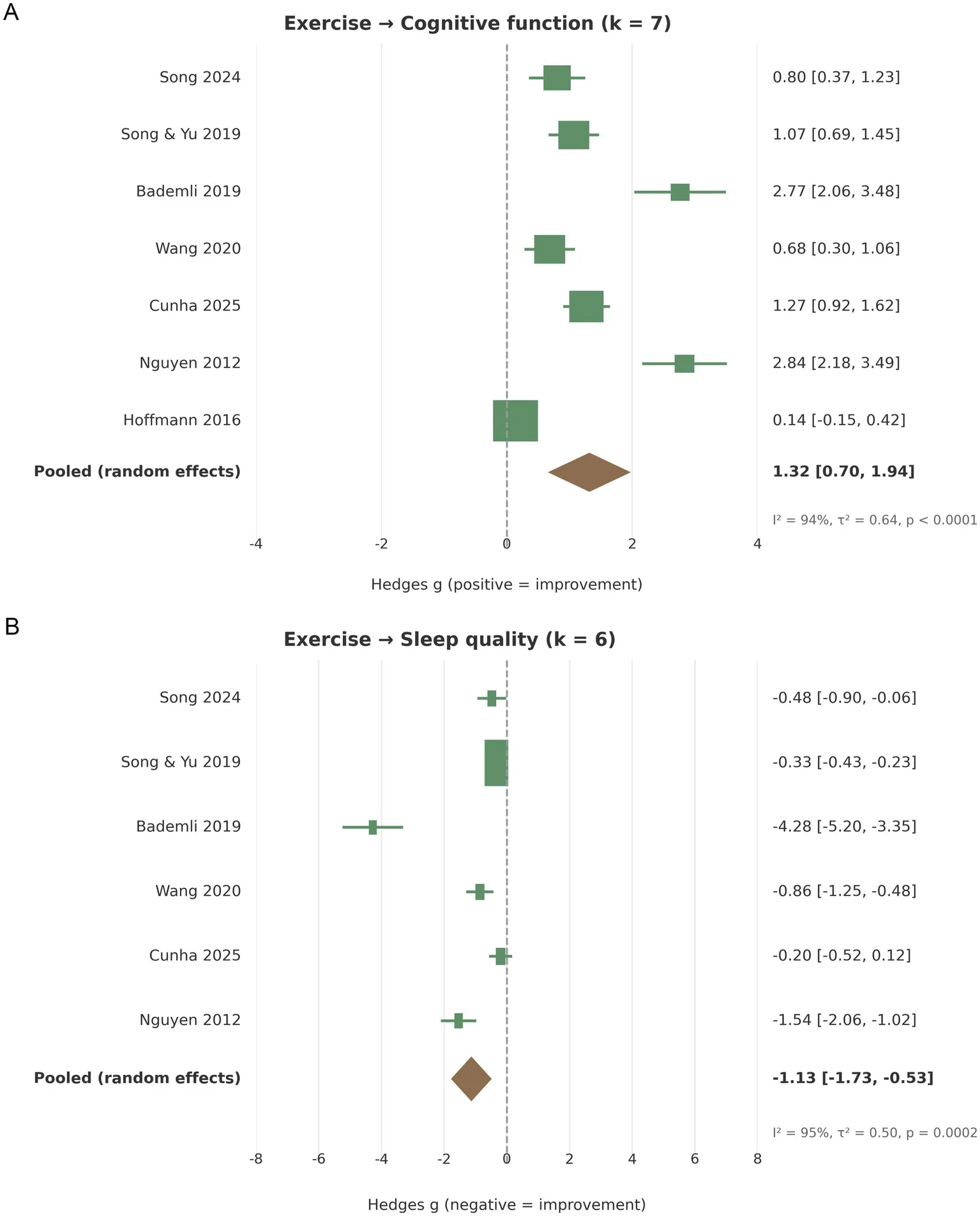
Forest plots of exercise effects on cognitive function and sleep quality. **(A)** Standardized mean differences (SMD) with 95% confidence intervals for cognitive function outcomes. The pooled effect size was SMD = 1.32 (95% CI: 0.70–1.94), indicating a large improvement in cognitive function following exercise interventions. Heterogeneity was substantial (*I*^2^ = 93.7%, τ^2^ = 0.64). **(B)** Standardized mean differences (SMD) with 95% confidence intervals for sleep quality outcomes. Negative SMD values indicate improvement in sleep quality. The pooled effect size was SMD = −1.13 (95% CI: −1.73 to −0.53), with high heterogeneity (*I*^2^ = 94.7%, *τ*^2^ = 0.50). In both panels, each square represents an individual study, with the size of the square proportional to the study weight in the random-effects model. The diamond represents the pooled effect estimate.

### 3.5 Effects of exercise on sleep quality

Exercise was found to significantly improve sleep quality, with a pooled standardised mean difference of −1.13 (95% CI: −1.73 to −0.53, *p* < 0.001). Negative values indicate improvement. However, heterogeneity was high (*I*^2^ = 94.7%, *τ*^2^ = 0.50; see Figure 3B). The effect was significant in participants with MCI (SMD = −1.34, 95% CI: −2.23 to −0.45) but not in the two trials of cognitively healthy older adults (SMD = −0.85, 95% CI: −2.16 to 0.46), and was larger for interventions lasting more than 16 weeks (SMD = −2.16) than for shorter programmes (SMD = −0.33). Including the sensitivity-only study (Karydaki et al., 2017) yielded a similar pooled effect (SMD = −1.00, 95% CI: −1.54 to −0.47). A sensitivity analysis excluding the extreme outlying effect (Bademli et al., 2019; SMD = −4.28) reduced the pooled effect to SMD = −0.63 (95% CI: −1.00 to −0.27; *I*^2^ = 85.4%). These findings build on existing evidence regarding the positive impact of exercise on sleep quality in older adults (Kredlow et al., 2015; Páez et al., 2024) and demonstrate that exercise has a beneficial effect on both cognitive function and sleep quality. This satisfies a prerequisite for mediation analysis.

### 3.6 Mediation analysis results

The meta-analytic structural equation model was estimated on the pooled correlation matrix of the six studies reporting complete exercise, sleep and cognitive data (*k* = 6; see Figure 4). Because the model is just-identified (saturated), global fit indices are not applicable; parameter uncertainty was instead quantified with 10,000 study-level bootstrap resamples. Path a (Exercise → Sleep) was significant (*β* = −0.473, *p* < 0.001), indicating that exercise improves sleep quality. Path b (Sleep → Cognition, controlling for exercise) was also significant (*β* = −0.633, *p* < 0.001), indicating that poorer sleep quality was associated with worse cognitive function. The indirect effect through sleep quality was *β* = 0.300 (95% bootstrap CI: 0.125–0.404, *p* < 0.001), accounting for 50.8% of the total effect (total effect *c* = 0.589, 95% CI: 0.397–0.732; proportion mediated = indirect effect/total effect × 100% = 0.300/0.589 × 100% = 50.8%; bootstrap 95% CI of the proportion: 24.7–71.8%). The direct effect (path c′) also remained significant (*β* = 0.290, *p* < 0.001), suggesting that sleep quality partially rather than completely mediates the relationship between exercise and cognition. To our knowledge, these results provide novel quantitative evidence that approximately half of the cognitive benefits of exercise for older adults are transmitted through improvements in sleep quality.

**Figure 4:**
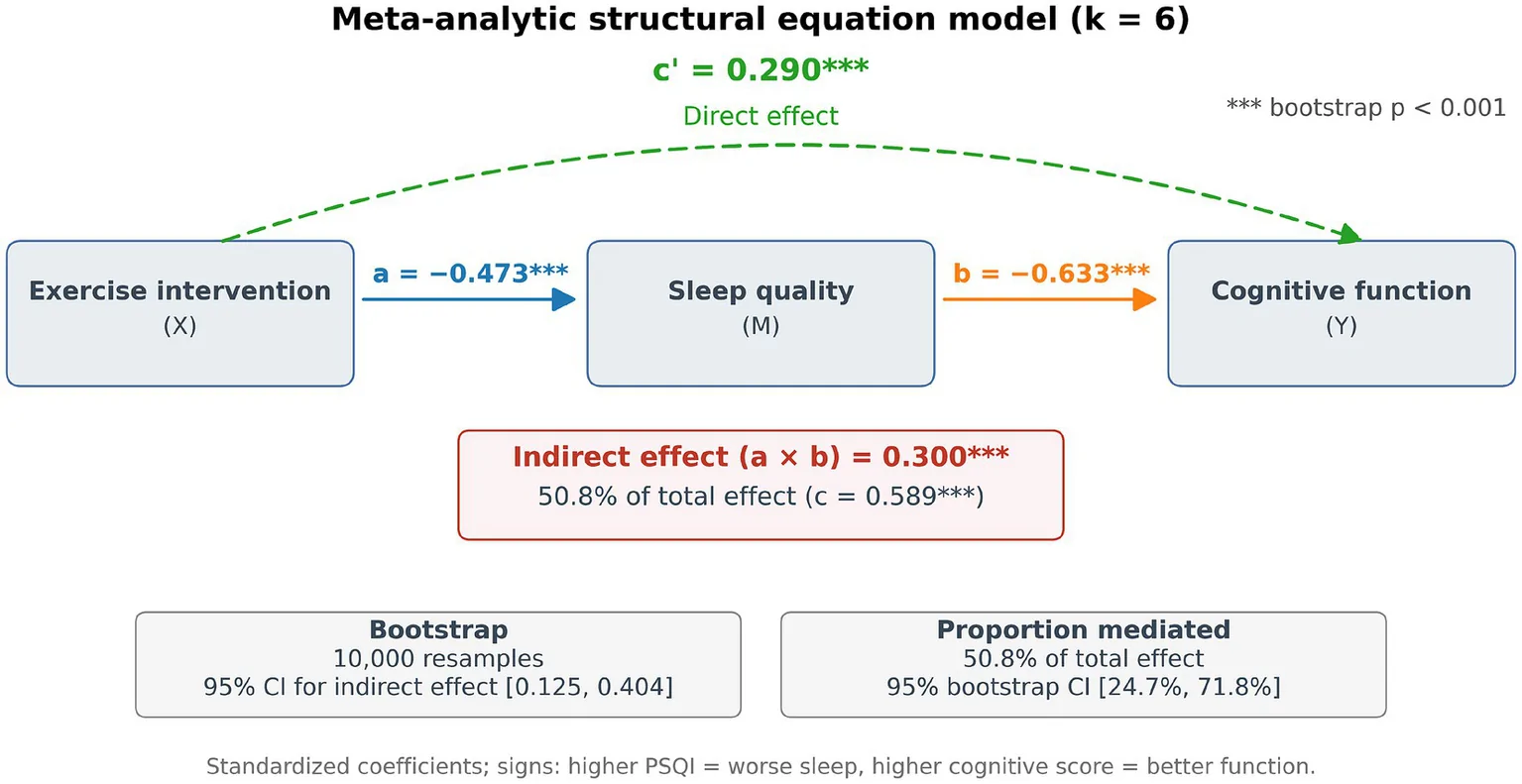
Meta-analytic structural equation model (MASEM) showing the mediation effect of sleep quality on the exercise–cognition relationship (*k* = 6 studies). Path coefficients are standardized *β* values estimated from the pooled correlation matrix. Path a (Exercise → Sleep): *β* = −0.473, *p* < 0.001. Path b (Sleep → Cognition, controlling for exercise): *β* = −0.633, *p* < 0.001. The indirect effect (a × b) was *β* = 0.300 (95% bootstrap *CI*: 0.125–0.404, 10,000 resamples), accounting for 50.8% (95% *CI*: 24.7–71.8%) of the total effect. The direct effect (path c′): *β* = 0.290, *p* < 0.001. Signs: higher PSQI scores indicate worse sleep; higher cognitive scores indicate better function. ***p < 0.001.

### 3.7 Moderators of the mediation effect

The proportion of the total cognitive effect mediated through sleep varied considerably across subgroups (see Table 2 and Figure 5). The mediation proportion was substantially larger in participants with mild cognitive impairment (67.6%; indirect effect *β* = 0.354, 95% CI: 0.132–0.498, *p* < 0.001) than in cognitively healthy older adults (31.9%; *β* = 0.223, 95% CI: 0.067–0.307, *p* < 0.001), although the latter estimate is exploratory because it rests on only two studies. The mediation proportion also increased with intervention duration, from 23.2% (*β* = 0.110, 95% CI: 0.072–0.169) for programmes of 16 weeks or less to 55.3% (*β* = 0.387, 95% CI: 0.181–0.492) for programmes exceeding 16 weeks (see Supplementary Figure S1). A subgroup sensitivity analysis excluding the study with the most extreme sleep effect (Bademli et al., 2019) yielded a smaller but still significant indirect effect (*β* = 0.205, 95% CI: 0.101–0.289; 38.7% mediated). Subgroup analyses by exercise type and by sleep-measurement modality were not performed, because each exercise category contained too few studies for reliable estimation and all studies assessed sleep with the self-reported PSQI. These moderator findings are summarized visually in Figure 5. Given the small number of studies within each stratum (as few as one), all subgroup contrasts should be interpreted as hypothesis-generating. Formal statistical tests for subgroup differences in the mediation proportion were not performed, because individual strata contained as few as one or two studies, which renders any interaction test severely underpowered and uninterpretable; we note, however, that the 95% bootstrap confidence intervals of the corresponding indirect effects overlapped (for example, 0.132–0.498 for mild cognitive impairment versus 0.067–0.307 for cognitively healthy participants), so the observed differences are descriptive rather than confirmatory.

**Table 2:** Subgroup and sensitivity analyses of the sleep-mediated effect.

Subgroup	*k* (studies)	*N* (participants)	Total effect c	Indirect effect *β* (95% CI)	Mediation proportion (%)	*p*-value (indirect)	Analysis set
Baseline cognitive status							
Mild Cognitive Impairment	4	380	0.524	0.354 (0.132–0.498)	67.6	<0.001	Main subgroup
Cognitively healthy	2	222	0.699	0.223 (0.067–0.307)	31.9	<0.001	Exploratory (*k* = 2)
Intervention duration							
≤16 weeks	3	358	0.474	0.110 (0.072–0.169)	23.2	<0.001	Main subgroup
>16 weeks	3	244	0.699	0.387 (0.181–0.492)	55.3	<0.001	Main subgroup
Sensitivity analyses							
Excluding Bademli et al. (2019)	5	542	0.531	0.205 (0.101–0.289)	38.7	<0.001	Sensitivity
Sleep–cognition *r* = −0.60	6	602	0.589	0.196 (0.079–0.241)	33.2	0.009	Sensitivity
Sleep–cognition *r* = −0.50	6	602	0.589	0.135	22.9	0.082	Sensitivity
Overall (pooled)	6	602	0.589	0.300 (0.125–0.404)	50.8	<0.001	Main MASEM

Total and indirect effects are standardized coefficients from the meta-analytic structural equation model (MASEM) estimated on pooled correlation matrices; confidence intervals and *p*-values are based on 10,000 study-level bootstrap resamples. *k*, number of studies; *N*, number of participants; MCI, mild cognitive impairment.

**Figure 5:**
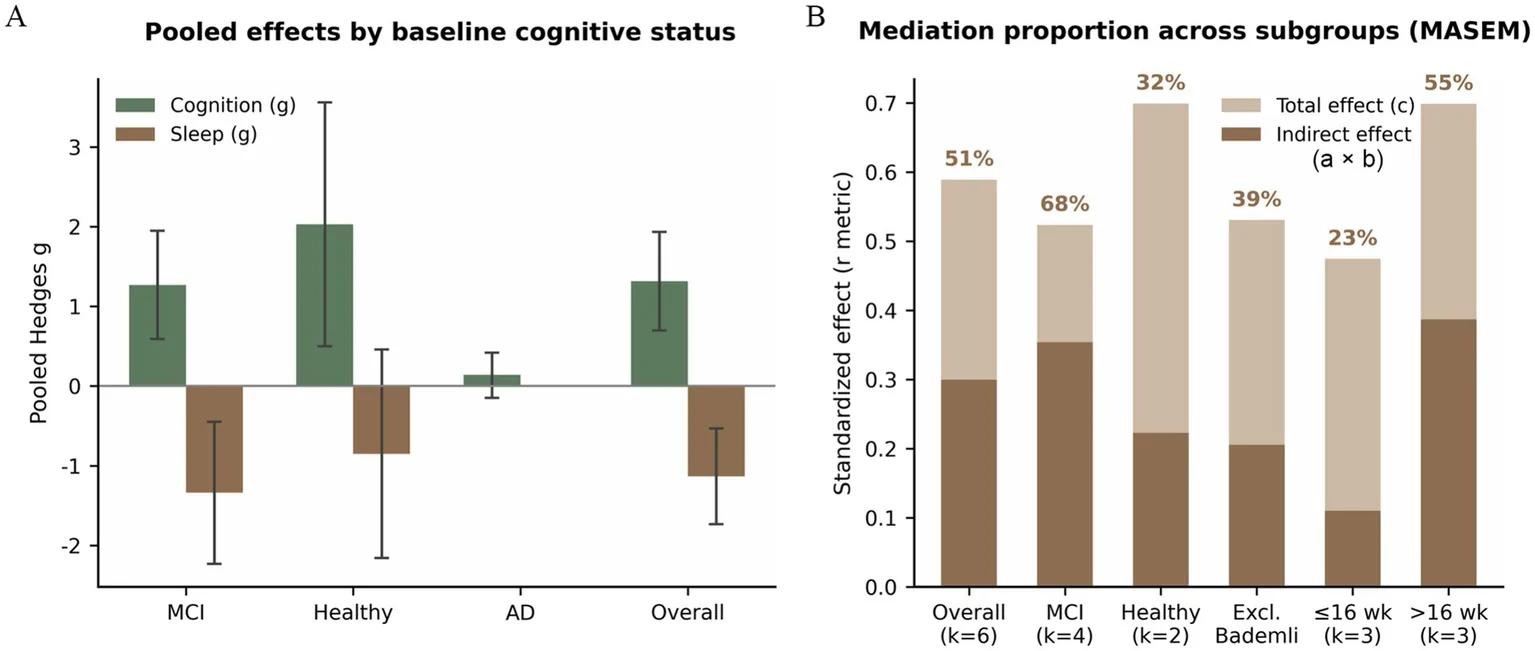
Subgroup analyses of mediation effects by population and intervention duration. Panels show the mediation proportion (percentage of the total exercise–cognition effect mediated through sleep improvement) for: **(A)** the overall pooled model (50.8%; indirect effect *β* = 0.300, 95% *CI*: 0.125–0.404); **(B)** baseline cognitive status (mild cognitive impairment: 67.6%, *β* = 0.354 [0.132–0.498]; cognitively healthy: 31.9%, *β* = 0.223 [0.067–0.307]); **(C)** Intervention duration (≤16 weeks: 23.2%, *β* = 0.110 [0.072–0.169]; >16 weeks: 55.3%, *β* = 0.387 [0.181–0.492]); and **(D)** A sensitivity analysis excluding the extreme sleep-effect outlier (Bademli et al., 2019: 38.7%, *β* = 0.205 [0.101–0.289]). Bars represent total effects and indirect (mediated) effects, with percentages indicating the proportion mediated. Error bars represent 95% bootstrap confidence intervals (10,000 resamples).

### 3.8 Sensitivity analyses and publication bias

Sensitivity analyses demonstrated the robustness of the main findings. Leave-one-out analyses confirmed that no single study drove either pooled effect (Supplementary Figure S5). Re-estimating both models with REML produced consistent point estimates with wider intervals (cognition: g = 1.33, 95% CI: 0.58 to 2.08, *p* < 0.001; sleep: *g* = −1.24, 95% CI: −2.42 to −0.06, *p* = 0.040), and all 95% prediction intervals crossed zero, reflecting the substantial heterogeneity (Supplementary Table S5). Adding the sensitivity-only study (Karydaki et al., 2017) to the sleep meta-analysis left the pooled effect essentially unchanged (SMD = −1.00 vs. −1.13). Excluding the study with the extreme sleep effect (Bademli et al., 2019) attenuated the pooled sleep effect (SMD = −0.63, 95% CI: −1.00 to −0.27) and the MASEM indirect effect (*β* = 0.205, 95% CI: 0.101–0.289; 38.7% mediated), but both remained statistically significant. Varying the single externally sourced sleep–cognition correlation (*r* = −0.77; Wang et al., 2020) to more conservative values (−0.60 and −0.50) reduced the indirect effect (*β* = 0.196, 95% CI: 0.079–0.241, and *β* = 0.135, respectively; 33.2 and 22.9% mediated); under the most conservative scenario the bootstrap confidence interval included zero in some resamples, indicating that the magnitude of the indirect effect scales with the strength of the sleep–cognition association. Funnel-plot inspection and Egger’s regression test suggested asymmetry for cognitive outcomes (intercept = 12.20, *p* = 0.005; see Supplementary Figure S3A) but not for sleep outcomes (intercept = −4.69, *p* = 0.083; see Supplementary Figure S3B), raising the possibility of small-study effects or publication bias for cognitive outcomes. However, with fewer than 10 studies per analysis the power of these tests is low, and the results should be interpreted with caution; trim-and-fill adjustment was therefore not performed, because the procedure is unreliable with so few studies. As a further sensitivity analysis, Hartung–Knapp adjustment widened the confidence intervals considerably (Model A: SMD = −1.13, 95% CI: −2.62 to 0.35, *p* = 0.107; Model B: SMD = 1.32, 95% CI: 0.37 to 2.26, *p* = 0.014); under this more conservative estimator the pooled sleep effect was no longer statistically significant at *k* = 6, whereas the cognitive effect remained significant, so the sleep-related findings should be interpreted with corresponding caution.

## 4 Discussion

This meta-analysis provides quantitative evidence that sleep quality significantly mediates the relationship between exercise and cognitive function in older adults. Our main findings show that exercise leads to large improvements in cognitive function (standardized mean difference (SMD) = 1.32) and sleep quality (SMD = −1.13), with around half (50.8%) of the total cognitive benefit being mediated by improved sleep. The mediating role of sleep was more pronounced in individuals with mild cognitive impairment (67.6%) than in cognitively healthy older adults (31.9%) and was stronger in interventions lasting more than 16 weeks (55.3%) than in shorter programmes (23.2%). These results highlight the importance of sleep as a mechanistic link and therapeutic target in optimizing exercise interventions for cognitive health in ageing.

The finding that approximately half of the total effect is mediated through sleep suggests a substantial, albeit partial, mechanism. The remaining share, attributable to the direct effects of exercise, is consistent with the multifactorial nature of the cognitive benefits of exercise, which include direct vascular, metabolic, and neurotrophic effects (Erickson et al., 2011; Voss et al., 2013). Three interconnected neurobiological mechanisms may underlie the sleep-mediated pathway (see Figure 6). Firstly, the glymphatic system, which is active during slow-wave sleep, removes *β*-amyloid and tau (Xie et al., 2013; Iliff et al., 2012; He et al., 2017; Liu et al., 2022). Exercise enhances this clearance function (He et al., 2017), and the improvements in sleep quality observed in the included trials are consistent with this mechanism. Secondly, exercise increases BDNF levels, which are consolidated during sleep-dependent memory processes (Vivar et al., 2013). Longer interventions (>16 weeks) showed stronger mediating effects, likely reflecting the time required for BDNF-mediated synaptic remodeling and preservation of hippocampal volume (Erickson et al., 2011; Erickson et al., 2009). Furthermore, animal studies demonstrate that exercise promotes hippocampal neurogenesis through growth factor signalling (Fabel et al., 2009), and that sleep is necessary for consolidating these changes. Thirdly, exercise reduces neuroinflammation by lowering IL-6 and TNF-α levels and reducing microglial activation. Improved sleep also suppresses nocturnal inflammatory processes (Irwin and Opp, 2016; Hu et al., 2024). This dual anti-inflammatory effect may be particularly beneficial in mild cognitive impairment (MCI), where elevated neuroinflammation contributes to disease progression. Taken together, these mechanisms imply that exercise improves sleep quality. This, in turn, facilitates glymphatic clearance, BDNF-mediated neuroplasticity, and the resolution of inflammation. Ultimately, this enhances cognitive function (Figure 6). It should be emphasised that none of these mechanisms was directly measured in the included trials; they are synthesised from prior experimental and animal studies and are presented as a conceptual framework (Figure 6) intended to aid interpretation and to generate hypotheses for future mechanistic research, rather than as evidence arising from the present meta-analysis.

**Figure 6:**
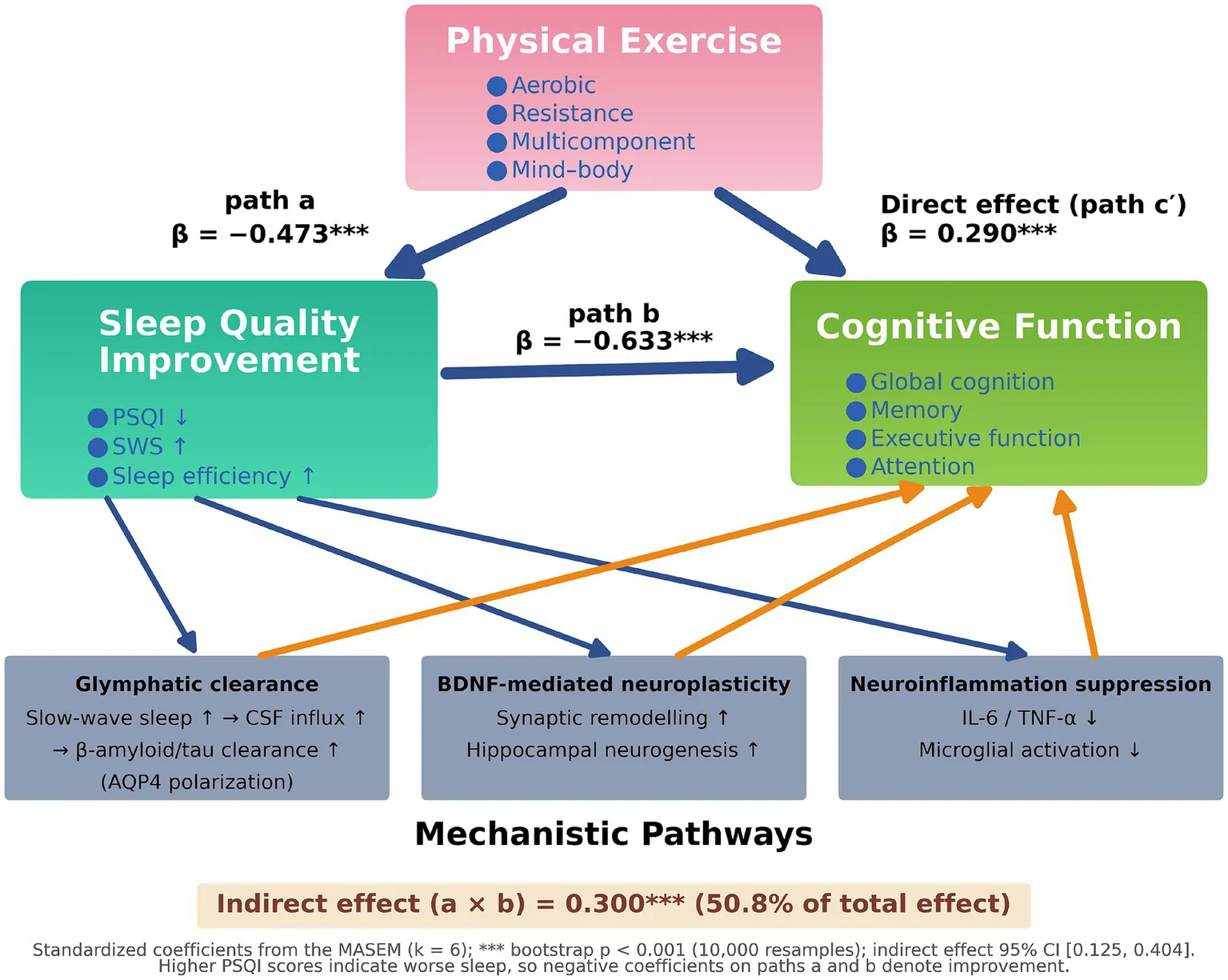
Theoretical framework of the sleep-mediated exercise—cognition pathway. The model integrates three interconnected neurobiological mechanisms: (1) glymphatic clearance—exercise enhances cerebrospinal fluid influx and aquaporin-4 polarization during slow-wave sleep, facilitating clearance of *β*-amyloid and tau proteins; (2) BDNF-mediated neuroplasticity—exercise upregulates brain-derived neurotrophic factor, which is consolidated during sleep-dependent memory processes and supports synaptic remodeling and hippocampal neurogenesis; and (3) neuroinflammation suppression—exercise attenuates microglial activation, while improved sleep suppresses nocturnal inflammatory processes. These mechanisms collectively contribute to enhanced cognitive function in older adults. Arrows indicate directional pathways. BDNF, brain-derived neurotrophic factor. This framework is conceptual: the mechanisms shown were not directly measured in the included trials and are synthesised from prior experimental literature to aid interpretation and hypothesis generation.

Our cognitive effect size (SMD = 1.32) is larger than the values reported in previous meta-analyses of exercise and cognition in older adults (Smith et al., 2010; Northey et al., 2018; Gomes-Osman et al., 2018; Barha et al., 2017). This may reflect our focus on trials that measured both sleep and cognition, the high prevalence of baseline sleep disturbance in the included populations, and the influence of small trials with large effects; the substantial heterogeneity (*I*^2^ > 90%) and the funnel-plot asymmetry for cognitive outcomes indicate that the pooled estimate should be interpreted with caution. Our sleep effect size (SMD = −1.13) is larger than the effects reported by Kredlow et al. (2015) for regular exercise across sleep domains (*d* = 0.17–0.74) in a wider age range, probably because we focused on older adults, most of whom had impaired sleep or cognition at baseline. No previous study has quantitatively estimated this mediated proportion, so direct comparison with earlier work is not possible; however, the substantial mediated share is consistent with qualitative syntheses proposing a tight, compensatory coupling between physical activity, sleep and cognition (Sewell et al., 2024), and suggests that sleep may be an especially prominent pathway in the cognitive domain. Because the MASEM is estimated on the pooled correlation matrix, between-study heterogeneity propagates into the indirect-effect estimate; we therefore quantified parameter uncertainty with 10,000 study-level bootstrap resamples rather than asymptotic standard errors and verified the consistency of the findings using REML re-estimation and prediction intervals (Supplementary Table S5). Nevertheless, the precision of the mediation proportions, particularly within subgroups, remains limited by the substantial heterogeneity and the small number of contributing studies.

Notably, all included studies assessed sleep with self-reported instruments (the PSQI in seven studies and a caregiver-rated inventory subdomain reported only in graphical form in one study); no eligible trial used objective sleep measures such as polysomnography or actigraphy, although the feasibility of objective sleep assessment in exercise studies of older adults has been demonstrated (Sewell et al., 2023). This has significant methodological implications: self-report measures such as the PSQI capture perceived sleep quality and distress but not sleep architecture, efficiency, or slow-wave sleep duration—parameters more directly linked to glymphatic clearance and neuroplasticity. The true mechanistic role of sleep in the exercise–cognition pathway may therefore be under- or over-estimated. This finding highlights the importance of incorporating objective sleep monitoring in future intervention studies designed to test mediation.

These findings have several practical implications. Firstly, sleep should be considered a co-primary outcome in exercise trials targeting cognitive function rather than a secondary endpoint. The FITT principle should be optimised to deliver cognitive and sleep benefits; our data suggest that programmes lasting more than 16 weeks yield the largest sleep-mediated cognitive benefits. Secondly, routine screening for sleep problems using validated instruments or objective monitoring could identify older adults who are most likely to benefit from the intervention, particularly those with mild cognitive impairment (MCI), enabling a more personalized approach. Thirdly, combined exercise and sleep hygiene interventions may have synergistic effects, which is consistent with the emerging 24-h movement guidelines (World Health Organization, 2020). Fourthly, objectively measured sleep should be incorporated into mechanistic intervention studies in order to accurately capture the pathways through which exercise confers cognitive benefits.

The relationship between exercise, sleep and cognition is likely to be bidirectional and dynamic (Sewell et al., 2024), which complicates simple mediation models and suggests that dynamic systems approaches may better capture their interrelationships. Further longitudinal studies with repeated measures are needed to establish temporal precedence and causality.

This study has several strengths. These include a quantitative mediation test using MASEM with bootstrap inference; a comprehensive search strategy across five databases; and pre-specified sensitivity analyses testing the robustness of the mediation effect. However, several limitations should also be acknowledged. Firstly, the number of eligible trials was small (*k* = 6–7 per analysis), which limited statistical power for subgroup and publication-bias tests and precluded meta-regression and trim-and-fill adjustment; consistent with this, Hartung–Knapp sensitivity analyses indicated that the pooled sleep effect is sensitive to estimator choice at this sample of trials, as corroborated by REML re-estimation (Supplementary Table S5). Secondly, heterogeneity was substantial (*I*^2^ > 90% in both main analyses), reflecting differences in populations, interventions and outcome measures; although random-effects models and sensitivity analyses were used throughout, the pooled estimates should be interpreted with caution. Thirdly, as most of the included studies were not designed to test mediation, MASEM with a pooled correlation matrix was required rather than individual participant data (IPD); critically, the sleep–cognition correlation (path b) could be sourced from only one study (Wang et al., 2020), so the indirect-effect estimate depends heavily on that single coefficient, although sensitivity analyses using more conservative values preserved a significant indirect effect. Fourthly, although every included trial was a randomized controlled trial with longitudinal pre/post measurements at the participant level, the mediation analysis was necessarily performed on aggregated study-level summary data rather than on individual longitudinal participant data. The estimated paths therefore describe associations between study-level intervention effects, and causal mediation at the individual level can only be established by prospective, mediation-designed RCTs with repeated within-person measurements. Fifthly, for one study (Cunha et al., 2025) post-test values were digitised from published figures with imputed standard deviations, and for another (Song and Yu, 2019) the sleep effect was derived from the reported GEE coefficient because the printed post-test means were internally inconsistent; both decisions may have introduced minor imprecision. Sixthly, sleep was assessed exclusively with self-report instruments, and funnel-plot asymmetry suggested possible publication bias for cognitive outcomes. Seventhly, the cognitive outcome pooled highly heterogeneous measures spanning global cognition (MMSE, MoCA), memory, executive function and attention. Following established practice in exercise–cognition meta-analyses (Smith et al., 2010; Northey et al., 2018), the primary cognitive outcome of each trial was combined into a single global effect, because the small number of included studies precluded informative domain-specific analyses; this pooling assumes a common underlying cognitive benefit of exercise and may mask domain-specific mediation patterns.

Based on these findings and limitations, future research should prioritise the following: (1) prospective RCTs designed to test mediation with repeated measurements of sleep and cognition; (2) objective sleep measures to capture changes in sleep architecture; (3) neuroimaging and biomarker studies to directly assess glymphatic clearance, BDNF and neuroinflammation as parallel mediators; (4) dose–response analyses to determine the optimal exercise parameters; (5) investigation of additional moderators, such as APOE genotype, sex, baseline sleep quality and circadian chronotype; (6) implementation research to translate these findings into clinical practice through the delivery of integrated exercise–sleep interventions in community settings.

## 5 Conclusion

This meta-analysis provides novel, albeit exploratory, quantitative evidence that sleep quality significantly mediates the relationship between exercise and cognition among older adults, accounting for approximately half (50.8%) of the total cognitive benefit. The mediating pathway was strongest in individuals with mild cognitive impairment and in interventions lasting more than 16 weeks. These findings identify sleep as a candidate mechanistic link and a promising therapeutic target for optimizing exercise interventions. Incorporating sleep assessment and enhancement strategies into exercise programmes is a promising way to maximize cognitive benefits and address the growing public health issue of age-related cognitive decline. Given the small number of eligible trials and the substantial heterogeneity, larger mediation-designed RCTs incorporating objective sleep measures are needed to confirm these estimates.

## Data Availability

The original contributions presented in the study are included in the article/Supplementary material, further inquiries can be directed to the corresponding author.
