# Supplementary material for: Effects of exercise on cognitive function in older adults: a systematic review and meta-analysis of the mediating role of sleep
Source: Front Hum Neurosci. 2026 Sep 28;20:1949679. doi: 10.3389/fnhum.2026.1949679 (PMC13654707; doi:10.3389/fnhum.2026.1949679)
Supplement: Supplementary file 1 [file Table_1.docx]

**Supplemental Tables**

**TABLE S1**  PRISMA 2020 Checklist.

| **Section/Topic** | **Item** | **Checklist Item** | **Reported on Page #** |
| --- | --- | --- | --- |
| **TITLE** |  |  |  |
| Title | 1 | Identify the report as a systematic review. | N/A |
| **ABSTRACT** |  |  |  |
| Abstract | 2 | Provide a structured summary including background, objectives, methods, results, and conclusions. | N/A |
| **INTRODUCTION** |  |  |  |
| Rationale | 3 | Describe the rationale for the review in the context of existing knowledge. | N/A |
| Objectives | 4 | Provide an explicit statement of the objective(s) or question(s) addressed by the review. | N/A |
| **METHODS** |  |  |  |
| Protocol | 5 | Indicate whether a review protocol exists and if and where it can be accessed (e.g., PROSPERO). | N/A |
| Eligibility criteria | 6 | Specify study characteristics and report characteristics used as criteria for inclusion. |  |
| Information sources | 7 | Describe all searches and dates of coverage. |  |
| Search strategy | 8 | Present full search strategies for all databases. |  |
| Selection process | 9 | State the process for selecting studies (screening, eligibility). |  |
| Data collection process | 10 | Describe method of data extraction and items sought. |  |
| Data items | 11 | List and define all outcomes for which data were sought. |  |
| Risk of bias | 12 | Specify methods for assessing risk of bias. |  |
| Effect measures | 13 | Specify for each outcome the effect measure(s) (e.g., SMD). |  |
| Synthesis methods | 14 | Describe processes used to synthesize results (e.g., random-effects models, MASEM). |  |
| Reporting bias | 15 | Describe methods for assessing publication bias. |  |
| Certainty assessment | 16 | Describe methods for assessing certainty of evidence. |  |
| **RESULTS** |  |  |  |
| Study selection | 17 | Describe results of search and selection process. |  |
| Study characteristics | 18 | Cite each included study and present its characteristics. |  |
| Risk of bias | 19 | Present assessments of risk of bias for each included study. |  |
| Results of individual studies | 20 | Present results for all outcomes (e.g., SMD with 95% CI). |  |
| Results of synthesis | 21 | Present results of meta-analyses and subgroup analyses. |  |
| Reporting bias | 22 | Present assessments of publication bias. |  |
| Certainty of evidence | 23 | Present assessments of certainty of evidence. |  |
| **DISCUSSION** |  |  |  |
| Interpretation | 24 | Provide interpretation considering objectives, limitations, and multiplicity. |  |
| Limitations | 25 | Discuss limitations of review evidence. |  |
| Conclusions | 26 | Provide general interpretation and implications. |  |

**TABLE S2**  Characteristics of Excluded Studies (N = 7)

| **No.** | **First Author, Year** | **Title** | **Reason for Exclusion** |
| --- | --- | --- | --- |
| 1 | Alessi et al., 1999 | A randomized trial of a combined physical activity and environmental intervention in nursing-home residents: do sleep and agitation improve? | Outcomes: No post-test cognitive outcome |
| 2 | Choi and Sohng, 2018 | The effects of a floor-seated exercise program on physical fitness, depression, and sleep in older adults: a cluster randomized controlled trial | Outcomes: No post-test between-group cognitive data |
| 3 | McCurry et al., 2011 | Increasing walking and bright light exposure to improve sleep in community-dwelling persons with Alzheimer's disease: results of a randomized, controlled trial | Outcomes: No post-test cognitive outcome |
| 4 | Nascimento et al., 2014 | Physical exercise in MCI elderly promotes reduction of pro-inflammatory cytokines and improvements on cognition and BDNF peripheral levels | Outcomes: No sleep outcome |
| 5 | Reid et al., 2010 | Aerobic exercise improves self-reported sleep and quality of life in older adults with insomnia | Outcomes: No post-test cognitive outcome |
| 6 | Siu et al., 2021 | Effects of Tai Chi or exercise on sleep in older adults with insomnia: a randomized clinical trial | Outcomes: No post-test cognitive outcome |
| 7 | Stella et al., 2011 | Attenuation of neuropsychiatric symptoms and caregiver burden in Alzheimer's disease by motor intervention: a controlled trial | Outcomes: Non-standard sleep measure and no standardised cognitive test |

*Note: This table lists all seven full-text reports excluded after eligibility assessment, with the primary reason for exclusion. Full-text screening was performed independently by two reviewers, with disagreements resolved by consensus.*

**TABLE S3** Summary of Exclusion Reasons

| **Exclusion Category** | **N** | **Percentage (%)** |
| --- | --- | --- |
| Outcomes: No post-test cognitive outcome | 4 | 57.1 |
| Outcomes: No sleep outcome | 1 | 14.3 |
| Outcomes: No post-test between-group cognitive data | 1 | 14.3 |
| Outcomes: Non-standard sleep measure and no standardised cognitive test | 1 | 14.3 |
| **Total** | **7** | **100.0** |

**TABLE S4**  Study-Level Correlations and Pooled Correlation Matrix Used in the MASEM

| **Study** | **N** | **Exercise–sleep correlation, r** | **Exercise–cognition correlation, r** |
| --- | --- | --- | --- |
| Song et al., 2024 | 89 | −0.234 | 0.372 |
| Song and Yu, 2019 | 120 | −0.162 | 0.471 |
| Bademli et al., 2019 | 60 | −0.906 | 0.810 |
| Wang et al., 2020 | 111 | −0.396 | 0.323 |
| Cunha et al., 2025 | 149 | −0.100 | 0.536 |
| Nguyen et al., 2012 | 73 | −0.609 | 0.817 |
| **Pooled (random effects, Fisher’s z)** | **602** | **−0.473 [−0.705, −0.150]** | **0.589 [0.397, 0.732]** |
| **Pooled correlation matrix** | **Exercise** | **Sleep quality** | **Cognitive function** |
| Exercise | 1.00 | — | — |
| Sleep quality | −0.473 | 1.00 | — |
| Cognitive function | 0.589 | −0.770ᵃ | 1.00 |

Note. Correlations are point-biserial correlations between the exercise intervention and each outcome, converted from study-level standardised mean differences. Pooled values were estimated with separate random-effects models using Fisher’s z transformation (DerSimonian–Laird); 95% confidence intervals are shown in brackets. ᵃ The sleep–cognition correlation could not be estimated from the included trials and was taken from the baseline correlation reported by Wang et al. (2020; r = −0.77, n = 111); sensitivity analyses used r = −0.60 and r = −0.50. Signs: higher PSQI scores indicate worse sleep quality; higher cognitive scores indicate better function. MASEM, meta-analytic structural equation modelling.

**TABLE S5** Sensitivity Analysis Using REML Estimation and 95% Prediction Intervals

| **Model** | **Estimator** | **τ²** | **Pooled g [95% CI]** | **p value** | **95% prediction interval** |
| --- | --- | --- | --- | --- | --- |
| Model B: cognition (k = 7) | DL (main) | 0.64 | 1.32 [0.70, 1.94] | <0.001 | −0.89 to 3.53 |
| Model B: cognition (k = 7) | REML | 0.97 | 1.33 [0.58, 2.08] | <0.001 | −1.39 to 4.05 |
| Model A: sleep (k = 6) | DL (main) | 0.50 | −1.13 [−1.73, −0.53] | <0.001 | −3.27 to 1.01 |
| Model A: sleep (k = 6) | REML | 2.10 | −1.24 [−2.42, −0.06] | 0.040 | −5.60 to 3.12 |

Note. DL, DerSimonian–Laird estimator (main analysis); REML, restricted maximum likelihood (sensitivity analysis). The 95% prediction interval estimates the range within which the true effect of a future comparable study is expected to fall, calculated as g ± t(k − 2) × √(τ² + SE(g)²). I² values are Q-based and identical across estimators (cognition 93.7%, sleep 94.7%). Under REML the pooled sleep effect remained statistically significant but with a substantially wider confidence interval and a larger τ², consistent with the Hartung–Knapp sensitivity analysis indicating that the sleep effect is sensitive to estimator choice at this small number of studies; the pooled cognitive effect was materially unchanged. All prediction intervals crossed zero, reflecting the substantial between-study heterogeneity.

**Supplemental Figures**

**FIGURE S1** Exploratory dose–response analysis by intervention duration. (A) Pooled Hedges g for cognitive function (positive values indicate improvement) and sleep quality (negative values indicate improvement) for interventions of ≤16 weeks versus >16 weeks. (B) Proportion of the total cognitive effect mediated through sleep improvement, estimated by MASEM: 23.2% (indirect effect β = 0.110, 95% CI: 0.072–0.169) for programmes of ≤16 weeks and 55.3% (β = 0.387, 95% CI: 0.181–0.492) for programmes of >16 weeks. Exploratory analysis with k = 3 studies per duration subgroup; error bars represent 95% confidence intervals.

**FIGURE S2**  Subgroup forest plots of exercise effects on cognitive function by baseline cognitive status. (A) Mild cognitive impairment (4 studies, N = 380): pooled SMD = 1.27 (95% CI: 0.59–1.95), with high heterogeneity (I² = 89.0%). (B) Cognitively healthy older adults (2 studies, N = 222): pooled SMD = 2.03 (95% CI: 0.50–3.56), with high heterogeneity (I² = 94.2%). (C) Alzheimer's disease (1 study, N = 190): SMD = 0.14 (95% CI: −0.15 to 0.42). In all panels, each square represents an individual study, with the size of the square proportional to the study weight in the random-effects model; diamonds represent pooled estimates; the vertical dashed line indicates the null effect (SMD = 0).

**FIGURE S3** Funnel plots for publication bias assessment. (A) Funnel plot of standard error by standardized mean difference (SMD) for cognitive function outcomes (k = 7). The vertical line represents the pooled effect estimate, and the diagonal lines represent the pseudo-95% confidence intervals around the pooled effect. Egger's regression test suggested funnel-plot asymmetry (intercept = 12.20, p = 0.005); however, with fewer than 10 studies the test has low power, and trim-and-fill adjustment was therefore not performed. (B) Funnel plot for sleep quality outcomes (k = 6). Egger's test was not significant (intercept = −4.69, p = 0.083). Each filled circle represents an individual study included in the meta-analysis. SMD, standardized mean difference; SE, standard error.

**FIGURE S4** Complete search strategies for all databases. Full Boolean search strings used in PubMed/MEDLINE, Web of Science, Cochrane CENTRAL, Embase, and PsycINFO from inception to June 2026.

**FIGURE S5** Leave-one-out sensitivity analyses of the pooled exercise effects. (A) Model B (cognition, k = 7); (B) Model A (sleep quality, k = 6). Each row shows the random-effects (DerSimonian–Laird) pooled Hedges g with 95% confidence interval and I² after omitting one study; the diamond shows the overall pooled effect across all studies. The pooled cognitive effect ranged from 1.07 (omitting Nguyen et al., 2012) to 1.52 (omitting Hoffmann et al., 2016), and the pooled sleep effect from −0.63 (omitting Bademli et al., 2019) to −1.38, with every iteration remaining statistically significant (all p < 0.01). Omitting Bademli et al. (2019) most attenuated the sleep effect and reduced heterogeneity (I² from 94.7% to 85.4%), consistent with its identification as an extreme-effect outlier; no single study altered the direction or statistical significance of either pooled effect.
